# Glycemic Index and Glycemic Load and Their Association with C-Reactive Protein and Incident Type 2 Diabetes

**DOI:** 10.1155/2011/623076

**Published:** 2011-05-05

**Authors:** Geertruida J. van Woudenbergh, Anneleen Kuijsten, Eric J. G. Sijbrands, Albert Hofman, Jacqueline C. M. Witteman, Edith J. M. Feskens

**Affiliations:** ^1^Division of Human Nutrition, Wageningen University, P.O. Box 8129, 6700 EV Wageningen, The Netherlands; ^2^Department of Internal Medicine, Erasmus Medical Center, Rotterdam, The Netherlands; ^3^Department of Epidemiology and Biostatistics, Erasmus Medical Center, Rotterdam, The Netherlands

## Abstract

*Objective*. To investigate whether the Glycemic Index (GI) or Glycemic Load (GL) of a diet is associated with C-reactive Protein (CRP) and risk of type 2 diabetes in a prospective study. *Materials and Methods*. Our analysis included 4,366 participants who did not have diabetes at baseline. During follow-up 456 diabetes cases were confirmed. Dietary GI and GL were derived from a food-frequency questionnaire and its association with CRP was examined cross-sectionally using linear regression models. The association of GI and GL with diabetes incidence was examined using Cox proportional hazard models. *Results*. GL, but not GI, was associated with lnCRP at baseline (*b*_GL_ = 0.11 per 50 units; *P* = .01). When comparing the highest to the lowest tertile of GI with respect to diabetes incidence, a Relative Risk (RR) of 0.95 [95%CI 0.75, 1.21] was found after adjustment for lifestyle and nutritional factors. For GL the RR for diabetes incidence was 1.00 [95%CI 0.74, 1.36]. Additional adjustment for CRP did not change RRs. *Conclusion*. Since GI was not associated with CRP and risk of type 2 diabetes, it is unlikely that a high GI diet induces the previously shown positive association between CRP and risk of type 2 diabetes by increasing CRP concentrations.

## 1. Introduction

A growing body of evidence suggests a role of low-grade chronic inflammation in the development of type 2 diabetes. C-reactive protein (CRP) is a physiological marker of inflammation and reflects chronic inflammation when the concentration of this marker is slightly elevated over a longer period of time [1]. A meta-analysis of 16 prospective cohort studies showed that CRP was associated with an increased risk of type 2 diabetes [2]. This risk may be attributed to central adiposity [2]. It is also suggested that elements of the diet, like Glycemic Index (GI) and Glycemic Load (GL), may play a role [3]. The GI expresses the influence of foods on blood glucose concentrations after consumption [4]. The GL makes allowance for the GI of a food product and the portion size eaten [5]. At least four cross-sectional studies showed a positive association of GI or GL with CRP [6–9]. GI and GL have also been related to an increased risk of type 2 diabetes in several cohort studies [10–14], but not in all [15–18]. So, GI or GL diets may be of importance in the development of type 2 diabetes, possibly due to its effect on CRP concentrations. 

We investigated, therefore, whether GI or GL is associated with CRP and subsequently with risk of type 2 diabetes in an elderly Dutch population. In this population a positive association between CRP and risk of type 2 diabetes was shown previously [19].

## 2. Materials and Methods

### 2.1. Study Population

**Figure 1:**
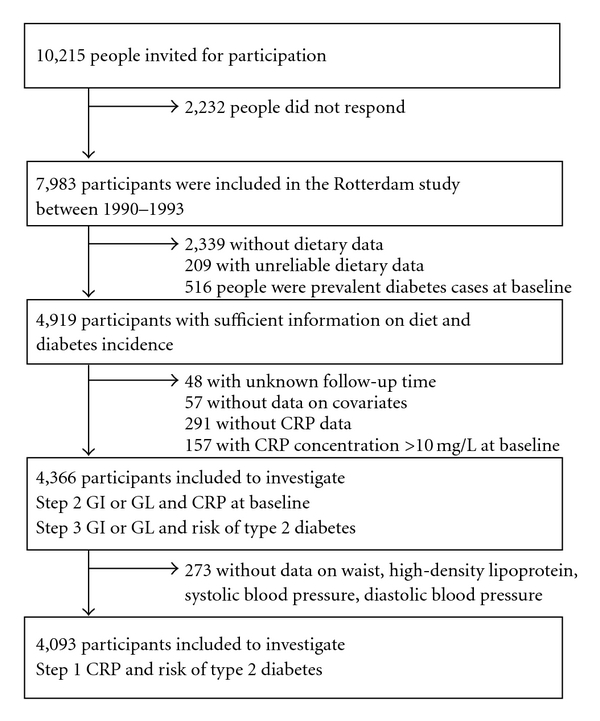
Flow diagram for selection of participants to investigate whether glycemic index (GI) or glycemic load (GL) is associated with C- reactive protein (CRP) and with risk of type 2 diabetes.

The Rotterdam study is a population-based prospective cohort study among inhabitants of Ommoord, a district of the city Rotterdam, The Netherlands [20]. In 1990 all inhabitants of this district who were aged ≥55 years were invited for participation (*n* = 10,215). Of the 7,983 responders (78%), 2,548 participants did not provide sufficient dietary data, 516 had type 2 diabetes at baseline, and 553 had not sufficient information on follow-up time or covariates (Figure 1). Hence, 4,366 participants were included in the current analysis. The Medical Ethics Committee of Erasmus Medical Center, Rotterdam, The Netherlands, approved the study. All participants gave informed consent.

### 2.2. Data Collection

#### 2.2.1. Glycemic Index and Glycemic Load

Dietary assessment at baseline comprised a self-administered questionnaire followed by a structured interview with a trained dietician at the research centre. Participants had to mark the foods and drinks they had consumed at least twice a month in the preceding year. Subsequently, the dietician obtained accurate information on the amount of food eaten using a semiquantitative food frequency questionnaire [21]. Intake of food items was converted into total intake of energy and nutrients using the Dutch Food Composition table 1993 (NEVO). For the intake of fiber we used the Dutch Food Composition table 1996 (NEVO). Validation of the questionnaire against 15 multiple-day food records in 80 participants showed a Pearson's correlation of 0.79 for adjusted intake of total carbohydrates [21]. 

To each single food product derived from the questionnaire, GI values were assigned. These values were based on international published GI tables [22, 23]. Average GI and GL values for each participant were calculated as follows:

(1) $$\begin{array}{cc} \text{Mean}\;\text{GI} & =\frac{\sum_{i=1}^{n}\left({\text{GI}}_{i}\times {\text{carbohydrates}}_{i}\right)}{\sum_{i=1}^{n}\left({\text{carbohydrates}}_{i}\right)},\\ \text{Mean}\;\text{GL} & =\frac{\sum_{i=1}^{n}\left({\text{GI}}_{i}\;\times {\text{carbohydrates}}_{i}\right)}{100}. \end{array}$$

GI*_i_* is the GI value of food product *i*. After mean GI and GL were calculated, mean GI and GL were adjusted for energy using the residual method [24].

#### 2.2.2. C-Reactive Protein

Nonfasting serum blood samples were collected at baseline. In the samples, high-sensitivity CRP was measured using a rate near-infrared particle immunoassay (Immage Immuno-chemistry System, Beckman Coulter, Fullerton, CA). The procedure has been described in more detail elsewhere [19]. CRP concentrations exceeding 10 mg/L at baseline were excluded from the analysis of CRP, because these higher concentrations reflect rather acute than chronic inflammation [1].

#### 2.2.3. Diabetes Incidence

*1990–1993 till 2005. *Participants were considered type 2 diabetes cases when they were registered by a general practitioner as having type 2 diabetes and had at least one of the following four criteria: plasma glucose concentration ≥7.0 mmol/L, random plasma glucose concentration ≥11.1 mmol/L, anti diabetes medication, treatment by diet. Diabetes cases were monitored until July 2005.

#### 2.2.4. Nondietary Covariates

General information, for example, smoking status, education level, family history of type 2 diabetes, was obtained with a questionnaire at baseline. A family history of type 2 diabetes was defined as having a parent, sibling, or both with diabetes onset between 30 and 65 years. A history of coronary heart diseases (CHD) was defined as a self-reported myocardial infarction or angina pectoris with hospital admission. Information on energy expenditure (kcal/d) was obtained during follow-up for 3,244 participants of our study population with a physical activity questionnaire (LASA Physical Activity Questionnaire) [25]. Consequently, energy expenditure could be used as measure of physical activity in those participants. Information on anthropometrics was obtained during a visit at the research centre at baseline. Waist circumference was measured at the level midway between the lower rib margin and the iliac crest with participants in standing position. Blood pressure was measured twice at the right brachial artery with a random-zero sphygmomanometer with the participant in a sitting position. The mean of two consecutive measurements was used. High density lipoprotein (HDL) was measured with HDL cholesterol assay (Roche Diagnostics) using polyethylene glycol-modified enzymes and dextran sulfate.

### 2.3. Statistical Analysis

Descriptive data were expressed as a mean (SD), a median (interquartile range), or a percentage. In order to investigate the effect of GI or GL on the association between CRP and type 2 diabetes, our analysis included three steps.

**Step 1**

The positive association between CRP and risk of type 2 diabetes, as shown previously in the Rotterdam Study (*n* = 5,901) [19], was verified in our subpopulation of the same study (*n* = 4,093) (Figure 1).

**Step 2**

Linear regression models were used with energy-adjusted GI or GL as independent variable and CRP at baseline as dependent variable. CRP was transformed logarithmically to achieve a normal distribution. In addition to energy-adjusted GI or GL, model 1 included age (years), sex, smoking (current, former, never), and family history of diabetes (yes, no) as covariates. Model 2 was similar to model 1 with additional adjustment for intake of five dietary factors: energy (kcal/d), protein (energy-%), saturated fat (energy-%), alcohol (0, >0–10, >10–20, >20 g/d), and fiber (g/d). Model 3 was similar to model 2 with additional adjustment for BMI (kg/m^2^).

**Step 3**

We explored the association between energy-adjusted GI or GL and risk of type 2 diabetes using Cox proportional hazard models. Hazard Ratios (RR) and 95% CI provided by these Cox models expressed the risk relative to the lowest tertile. Model 1, model 2, and model 3 included the same covariates as used in Step 2. To investigate a potential intermediate effect of CRP within the association of GI or GL with type 2 diabetes, an additional model was used. This model was similar to model 3 with additional adjustment for baseline lnCRP concentration.

In Steps 1 and 3, we modeled the median value of each tertile of GI or GL as continuous variable to test for linear trends across categories. To investigate potential effect measure modification, the association between GI and GL and risk of type 2 diabetes was studied separately for men and women and for participants with a low and high BMI (median split: ≤25.9 versus >25.9 kg/m^2^, resp.). 

Analyses were carried out using the statistical software program SAS version 9.1. A two-sided *P* value less than  .05 was considered as statistically significant for all analysis.

## 3. Results

At baseline, the mean of GI was 59 (SD 3) and mean GL was 127 (SD 22). The highest tertile of GI included more smokers and more men than the lowest tertile (Table 1). Intake of polysacharides increased, whereas intake of mono- and disaccharides and fiber decreased across tertiles of GI. Using stepwise regression, the main contributors to the variation in energy-adjusted GL appeared to be sweets (26%), fats (9%), bread (9%), alcoholic drinks (7%), and nuts (5%). The main contributors to the variation in energy-adjusted GI were milk products (28%), fruit (20%), bread (13%), potatoes (5%), and cakes (2%). Median CRP concentration was 1.65 mg/L, and 1,097 (25%) participants had an elevated CRP level (>3 mg/L) at baseline. 

Step 1 of our analysis included 4,093 participants of whom 423 developed type 2 diabetes during a median follow-up time of 11.0 years. The analysis confirmed that CRP at baseline was associated with an increased risk of type 2 diabetes after adjustment for age, sex, BMI, waist, systolic blood pressure, diastolic blood pressure, and HDL (RR_CRP  Q4  versus  Q1_: 1.76 (95%CI 1.27, 2.45); *P*_trend_ < .01). This RR was in line with RRs found when adjusted additionally for GI or GL (RR_CRP  Q4  versus  Q1_: 1.76 (95%CI 1.27, 2.43); RR_CRP  Q4  versus  Q1_: 1.76 (95%CI 1.27, 2.44), resp.). The association did not differ considerably between participants with a low or high GI diet (*P*_interaction_ = .53) and between participants with a low or high GL diet (*P*_interaction_ = .99). 

Step 2 of our analysis showed that after adjustment for lifestyle factors, nutritional factors, and BMI, a 50 unit increase in GL was associated with a 12% higher CRP concentration at baseline (*P* = .01) (Table 2). No association was observed for a 10 unit increase in GI (*b* = 0, *P* = .90). 

Step 3 of our analysis included 4,366 participants whose median follow-up was 12.4 years. A number of 456 participants developed type 2 diabetes. When comparing the highest to the lowest tertile in this population, an adjusted RR of 0.95 (95%CI 0.75, 1.21) was found for GI (model 3) (Table 3). For GL this adjusted RR_T_3_  versus  T_1__ was 1.00 (95%CI 0.74, 1.36). So, GI and GL were not associated with the risk of type 2 diabetes in this study. The RR found for GL was comparable with the one found for the association between intake of total carbohydrates and risk of type 2 diabetes (*Model 3 *RR_Q4  versus  Q1_: 1.04 (95%CI 0.71, 1.53)). The similar results also reflect the high correlation between total carbohydrates and GL (*r* = 0.93). After adding CRP at baseline to model 3, RRs for risk of type 2 diabetes did not change considerably (RR_GI  T_3_  versus  T_1__: 0.96 (95%CI 0.75, 1.22); RR_GL  T_3_  versus  T_1__: 0.99 (95%CI 0.73, 1.35)) (Table 3). In those with available information on physical activity, additional adjustment for energy expenditure did not change the RRs considerably (data not shown).

The association between GI or GL and risk of type 2 diabetes did not differ considerably between men and women (Model 3: *P*_interaction  GI_ = .75; *P*_interaction  GL_ = .08) and between participants with a low and high BMI (Model 3: *P*_interaction  GI_ = .32; *P*_interaction  GL_ = .29). Exclusion of participants with CHD at baseline (*n* = 514) did not substantially change the results (*Model 3 *RR_GI  T_3_  versus  T_1__: 0.93 (95%CI 0.72, 1.21); RR_GL  T_3_  versus  T_1__: 1.03 (95%CI 0.74, 1.43)).

## 4. Discussion

In this Dutch population, GL was associated positively with CRP at baseline, but not with risk of type 2 diabetes. GI was not associated with CRP nor with risk of type 2 diabetes. A high GI diet, therefore, could not explain the positive association between CRP and risk of type 2 diabetes by increasing CRP concentrations. 

We were able to study how GI and GL were associated with CRP and type 2 diabetes in a prospective cohort study with a high response rate, with a long follow-up period, with confirmed diabetes cases, and with available information on CRP concentration at baseline of a large population. 

Our FFQ measured adequately intake of carbohydrates, which was correlated highly with GL, but was not designed to measure GI or GL. It could be, therefore, that food products with a very high or low GI were not taken into account. This might explain the small range in GI and GL in our study. A comparable range in GI, however, was also observed in other Dutch cohorts in whom another FFQ was used [8, 26]. In one of the cohorts even a smaller range was found when GI was based on twelve 24-hr recalls instead of on a FFQ [26]. This shows that a small range in GI may exist in the Netherlands. National data on GI values of Dutch food products, however, would have provided more accurate measure of GI. 

GL, but not GI, was associated positively with CRP at baseline in this study. Due to the high correlation between GL and intake of carbohydrates in our population, the effect of GL itself could not be separated from the effect of total carbohydrate intake. Other cross-sectional studies on the association with CRP observed either positive associations for high GL diet [6] or high GI diet [7–9] or no association for GI [6, 27–29] or GL [7–9, 28–30]. No associations were also observed between changes in GI or GL and changes in CRP in a longitudinal study with a one-year follow-up [27]. On the contrary, one randomized trial in participants with type 2 diabetes showed that reduction in CRP concentration after 1 year was more pronounced in a low GI diet than a high GI diet [31]. Other randomized trials with a shorter duration, however, did not observe differential effects on CRP between a low GI diet and a high GI diet independently of weight lost [32–37]. Taking these results together, it is not very likely that GI affects CRP concentrations. The high within person variation in CRP, however, could have reduced the power of the statistical tests of the beta-coefficient [38, 39]. Therefore, duplicate measures of CRP should be used in new studies.

Our findings concerning the association of GI or GL with risk of type 2 diabetes are not in line with the conclusion of a meta-analysis published in 2008 [10]. This meta-analysis on five cohort studies showed that high GI or GL diets were associated with an increased risk of type 2 diabetes (RR_GI_ 1.40 (95%CI 1.23, 1.59); RR_GL_ 1.27 (95%CI 1.12, 1.45)). After this meta-analysis, these associations were investigated additionally in eight cohort studies. These studies found associations of GI with risk of type 2 diabetes ranging from a 6% lower risk to a 50% higher risk [11, 13–18]; with two of them statistically significant [11, 13]. The associations of GL with risk of type 2 diabetes ranged from a 20% lower risk to a 41% higher risk [11–17]. Three studies reported that their findings were statistically significant in women [12, 13] or in both sexes [14]. Four of these newly published studies [12–15] and our study met the inclusion criteria used in the meta-analysis by Barclay et al. [10]. Since ranges in GI do not always overlap among studies, a new pooled risk estimate would be difficult to interpret. Studies with high GI values (median of lowest category >63) observed higher risks of type 2 diabetes [5, 40, 41], whereas studies with low GI values (median of highest category <63) did not observe associations with type 2 diabetes [14, 15]. Our study gives additional information about the association between lower ranges of GI values and risk of type 2 diabetes, which was lacking in the meta-analysis. Overall, this might suggest that only high GI values are associated adversely with risk of type 2 diabetes.

## 5. Conclusion

Both GI and GL were not associated with risk of type 2 diabetes, although GL was associated positively with CRP concentrations. It is, therefore, unlikely that a high GI diet induces the positive association between CRP and risk of type 2 diabetes by increasing CRP concentrations.

## Figures and Tables

**Table 1:** Baseline characteristics of 4,366 Dutch adults aged ≥55 years by tertiles of energy-adjusted glycemic index (GI).^1^

	Low GI (<57.6)	Moderate GI (57.6–<60.3)	High GI (≥60.3)
	(*n* = 1,455)	(*n* = 1,456)	(*n* = 1,455)
Age (years)	67.3 (7.9)	67.7 (7.7)	66.9 (7.4)
Sex (% male)	26.6	39.7	54.5
Body Mass Index (kg/m^2^)	26.5 (3.6)	26.2 (3.4)	26.0 (3.8)
C-reactive protein (mg/L)^2^	1.6 (0.80–2.9)	1.7 (0.83–3.0)	1.7 (0.83–3.1)
Anti-inflammatory medication (%)	8.0	6.6	7.7
Family history of diabetes (%)	26.5	26.8	29.7
History of CHD (%)	10.0	11.9	13.4
Smoking (% current)	15.8	20.4	32.0
Education level (% low)	33.6	33.5	35.7
*Dietary intake*			
Total energy (kcal/d)	1967 (555)	2005 (491)	1971 (464)
Carbohydrate (en%)	44.2 (6.6)	44.7 (6.6)	43.7 (7.6)
Mono- and disacharides (en%)	24.5 (5.7)	22.6 (5.5)	19.7 (6.5)
Polysacharides (en%)	19.7 (3.7)	22.0 (3.7)	23.7 (4.4)
Energy-adjusted glycemic load	119 (19.0)	128 (20.1)	133 (23.1)
Fiber (g/d)	27.1 (8.3)	26.3 (6.4)	25.1 (6.6)
Protein (en%)	18.0 (3.3)	16.7 (2.8)	16.2 (2.8)
Fat (en%)	35.9 (6.3)	36.5 (6.1)	37.3 (6.2)
Saturated fatty acids (en%)	14.3 (3.4)	14.4 (3.1)	14.6 (3.1)
Mono-unsaturated fatty acids (en%)	12.3 (2.7)	12.3 (2.7)	12.6 (2.8)
Poly-unsaturated fatty acids (en%)	6.5 (2.8)	7.0 (2.8)	7.3 (2.9)
Alcohol drinkers (%)	79.2	82.2	79.9
Alcohol (g/d)^2,3^	6.0 (1.3–15.7)	6.4 (1.4–16.3)	8.7 (1.8–21.8)

^1^Means (SD) or percentages unless otherwise indicated.

^2^Values are expressed as median (interquartile range), because of their skewed distribution.

^3^Only in alcohol drinkers.

**Table 2:** Beta coefficients (SEE) for the association of energy-adjusted glycemic index (GI) or glycemic load (GL) with ln-C-reactive protein (CRP) in Dutch adults aged ≥55 years.

	Ln-CRP at baseline	
	*n* = 4,366	
	*GI per 10 units*	*GL per 50 units*
Crude model	0.11 (0.04), *P* < .01	−0.04 (0.03), *P* = .25
Model 1	0.04 (0.04), *P* = .31	−0.03 (0.03), *P* = .41
Model 2	0.05 (0.04), *P* = .29	0.09 (0.05), *P* = .05
Model 3	0.005 (0.04), *P* = .90	0.11 (0.04), *P* = .01

Values are beta-coefficients with SEE.

Model 1: adjusted for age, sex, smoking, and family history of diabetes.

Model 2: as model 1 with additional adjustments for intake of energy, protein, saturated fat, alcohol, and fiber.

Model 3: as model 2 with additional adjustment for BMI.

**Table 3:** Relative risks (95%CI) for incident type 2 diabetes by tertiles of energy-adjusted glycemic index and glycemic load in 4,366 Dutch adults aged ≥55 years.

	Low	Moderate	High	*P* for trend
Glycemic index	(<57.6)	(≥57.6–<60.3)	(≥60.3)	
Median	55.7	58.9	62.1	
# cases/total	149/1,455	141/1,456	166/1,455	
Person years	16,227	16,023	15,691	
Crude model	1	0.96 (0.76, 1.21)	1.16 (0.93, 1.44)	.19
Model 1	1	0.91 (0.72, 1.15)	1.02 (0.81, 1.29)	.84
Model 2	1	0.96 (0.76, 1.22)	1.06 (0.83, 1.35)	.64
Model 3	1	0.94 (0.74, 1.19)	0.95 (0.75, 1.21)	.71
Model 4	1	0.92 (0.73, 1.17)	0.96 (0.75, 1.22)	.75
Glycemic load	(<117.6)	(≥117.6–<134.6)	(≥134.6)	
Median	107.1	126.4	146.1	
# cases/total	173/1,455	149/1,456	134/1,455	
Person years	15,825	16,206	15,910	
Crude model	1	0.83 (0.67, 1.03)	0.77 (0.61, 0.96)	.02
Model 1	1	0.86 (0.69, 1.07)	0.77 (0.61, 0.96)	.02
Model 2	1	0.92 (0.72, 1.17)	0.98 (0.72, 1.33)	.86
Model 3	1	0.91 (0.71, 1.16)	1.00 (0.74, 1.36)	.96
Model 4	1	0.90 (0.70, 1.15)	0.99 (0.73, 1.35)	.91

Model 1: adjusted for age, sex, smoking, and family history of diabetes.

Model 2: as model 1 with additional adjustments for intake of energy, protein, saturated fat, alcohol, and fiber.

Model 3: as model 2 with additional adjustment for BMI.

Model 4: as model 3 with additional adjustment for ln C-reactive protein.

## References

[1] Pearson TA, Mensah GA, Alexander RW (2003). Markers of inflammation and cardiovascular disease: application to clinical and public health practice: a statement for healthcare professionals from the centers for disease control and prevention and the American Heart Association. *Circulation*.

[2] Lee CC, Adler AI, Sandhu MS (2009). Association of C-reactive protein with type 2 diabetes: prospective analysis and meta-analysis. *Diabetologia*.

[3] Browning LM, Jebb SA (2006). Nutritional influences on inflammation and type 2 diabetes risk. *Diabetes Technology and Therapeutics*.

[4] Jenkins DJA, Wolever TMS, Taylor RH (1981). Glycemic index of foods: a physiological basis for carbohydrate exchange. *American Journal of Clinical Nutrition*.

[5] Salmeron J, Manson JE, Stampfer MJ, Colditz GA, Wing AL, Willett WC (1997). Dietary fiber, glycemic load, and risk of non-insulin-dependent diabetes mellitus in women. *Journal of the American Medical Association*.

[6] Liu S, Manson JE, Buring JE, Stampfer MJ, Willett WC, Ridker PM (2002). Relation between a diet with a high glycemic load and plasma concentrations of high-sensitivity C-reactive protein in middle-aged women. *American Journal of Clinical Nutrition*.

[7] Levitan EB, Cook NR, Stampfer MJ (2008). Dietary glycemic index, dietary glycemic load, blood lipids, and C-reactive protein. *Metabolism*.

[8] Du H, van der A DL, van Bakel MME (2008). Glycemic index and glycemic load in relation to food and nutrient intake and metabolic risk factors in a Dutch population. *American Journal of Clinical Nutrition*.

[9] Qi L, van Dam RM, Liu S, Franz M, Mantzoros C, Hu FB (2006). Whole-grain, bran, and cereal fiber intakes and markers of systemic inflammation in diabetic women. *Diabetes Care*.

[10] Barclay AW, Petocz P, McMillan-Price J (2008). Glycemic index, glycemic load, and chronic disease risk—a metaanalysis of observational studies. *American Journal of Clinical Nutrition*.

[11] Krishnan S, Rosenberg L, Singer M (2007). Glycemic index, glycemic load, and cereal fiber intake and risk of type 2 diabetes in US black women. *Archives of Internal Medicine*.

[12] Hopping BN, Erber E, Grandinetti A, Verheus M, Kolonel LN, Maskarinec G (2010). Dietary fiber, magnesium, and glycemic load alter risk of type 2 diabetes in a multiethnic cohort in Hawaii. *Journal of Nutrition*.

[13] Villegas R, Liu S, Gao YT (2007). Prospective study of dietary carbohydrates, glycemic index, glycemic load, and incidence of type 2 diabetes mellitus in middle-aged Chinese women. *Archives of Internal Medicine*.

[14] Sluijs I, van der Schouw YT, van der A DL (2010). Carbohydrate quantity and quality and risk of type 2 diabetes in the European Prospective Investigation into Cancer and Nutrition-Netherlands (EPIC-NL) study. *American Journal of Clinical Nutrition*.

[15] Mosdol A, Witte DR, Frost G, Marmot MG, Brunner EJ (2007). Dietary glycemic index and glycemic load are associated with highdensity-lipoprotein cholesterol at baseline but not with increased risk of diabetes in the Whitehall II study. *American Journal of Clinical Nutrition*.

[16] Sahyoun NR, Anderson AL, Tylavsky FA, Lee JS, Sellmeyer DE, Harris TB (2008). Dietary glycemic index and glycemic load and the risk of type 2 diabetes in older adults. *American Journal of Clinical Nutrition*.

[17] Schulz M, Liese AD, Fang F, Gilliard TS, Karter AJ (2006). Is the association between dietary glycemic index and type 2 diabetes modified by waist circumference?. *Diabetes Care*.

[18] Barclay AW, Flood VM, Rochtchina E, Mitchell P, Brand-Miller JC (2007). Glycemic index, dietary fiber, and risk of type 2 diabetes in a cohort of older Australians. *Diabetes Care*.

[19] Dehghan A, Kardys I, de Maat MPM (2007). Genetic variation, C-reactive protein levels, and incidence of diabetes. *Diabetes*.

[20] Hofman A, Breteler MMB, van Duijn CM (2009). The rotterdam study: 2010 objectives and design update. *European Journal of Epidemiology*.

[21] Klipstein-Grobusch K, den Breeijen JH, Goldbohm RA (1998). Dietary assessment in the elderly: validation of a semiquantitative food frequency questionnaire. *European Journal of Clinical Nutrition*.

[22] Foster-Powell K, Holt SHA, Brand-Miller JC (2002). International table of gylcemic index and glycemic load values: 2002. *American Journal of Clinical Nutrition*.

[23] Atkinson FS, Foster-Powell K, Brand-Miller JC (2008). International tables of glycemic index and glycemic load values: 2008. *Diabetes Care*.

[24] Willett WC, Howe GR, Kushi LH (1997). Adjustment for total energy intake in epidemiologic studies. *American Journal of Clinical Nutrition*.

[25] Stel VS, Smit JH, Pluijm SMF, Visser M, Deeg DJH, Lips P (2004). Comparison of the LASA physical activity questionnaire with a 7-day diary and pedometer. *Journal of Clinical Epidemiology*.

[26] Du H, van der A DL, van Bakel MME, Verberne LDM, Ocke M, Feskens EJM (2009). Reproducibility and relative validity of dietary glycaemic index and glycaemic load assessed by the food-frequency questionnaire used in the dutch cohorts of the european prospective investigation into cancer and nutrition. *British Journal of Nutrition*.

[27] Griffith JA, Ma Y, Chasan-Taber L (2008). Association between dietary glycemic index, glycemic load, and high-sensitivity C-reactive protein. *Nutrition*.

[28] Beulens JWJ, de Bruijne LM, Stolk RP (2007). High dietary glycemic load and glycemic index increase risk of cardiovascular disease among middle-aged women. A population-based follow-up study. *Journal of the American College of Cardiology*.

[29] Huffman KM, Orenduff MC, Samsa GP, Houmard JA, Kraus WE, Bales CW (2007). Dietary carbohydrate intake and high-sensitivity C-reactive protein in at-risk women and men. *American Heart Journal*.

[30] Murakami K, Sasaki S, Takahashi Y (2008). Total n-3 polyunsaturated fatty acid intake is inversely associated with serum C-reactive protein in young Japanese women. *Nutrition Research*.

[31] Wolever TMS, Gibbs AL, Mehling C (2008). The Canadian Trial of Carbohydrates in Diabetes (CCD), a 1-y controlled trial of low-glycemic-index dietary carbohydrate in type 2 diabetes: no effect on glycated hemoglobin but reduction in C-reactive protein. *American Journal of Clinical Nutrition*.

[32] Hartman TJ, Albert PS, Zhang Z (2010). Consumption of a legume-enriched, low-glycemic index diet is associated with biomarkers of insulin resistance and inflammation among men at risk for colorectal cancer. *Journal of Nutrition*.

[33] Iannuzzi A, Licenziati MR, Vacca M (2009). Comparison of two diets of varying glycemic index on carotid subclinical atherosclerosis in obese children. *Heart and Vessels*.

[34] Jenkins DJA, Kendall CWC, McKeown-Eyssen G (2008). Effect of a low-glycemic index or a high-cereal fiber diet on type 2 diabetes: a randomized trial. *Journal of the American Medical Association*.

[35] Pittas AG, Roberts SB, Das SK (2006). The effects of the dietary glycemic load on type 2 diabetes risk factors during weight loss. *Obesity*.

[36] Shikany JM, Phadke RP, Redden DT, Gower BA (2009). Effects of low- and high-glycemic index/glycemic load diets on coronary heart disease risk factors in overweight/obese men. *Metabolism*.

[37] Botero D, Ebbeling CB, Blumberg JB (2009). Acute effects of dietary glycemic index on antioxidant capacity in a nutrient-controlled feeding study. *Obesity*.

[38] Feskens EJM, Bowles CH, Kromhout D (1991). Intra- and interindividual variability of glucose tolerance in an elderly population. *Journal of Clinical Epidemiology*.

[39] Macy EM, Hayes TE, Tracy RP (1997). Variability in the measurement of C-reactive protein in healthy subjects: implications for reference intervals and epidemiological applications. *Clinical Chemistry*.

[40] Salmeron J, Ascherio A, Rimm EB (1997). Dietary fiber, glycemic load, and risk of NIDDM in men. *Diabetes Care*.

[41] Schulze MB, Liu S, Rimm EB, Manson JE, Willett WC, Hu FB (2004). Glycemic index, glycemic load, and dietary fiber intake and incidence of type 2 diabetes in younger and middle-aged women. *American Journal of Clinical Nutrition*.

